# Implementation of an ISO15189 accredited next-generation sequencing service with the fully automated Ion Torrent Genexus: the experience of a clinical diagnostic laboratory

**DOI:** 10.1136/jcp-2022-208625

**Published:** 2022-12-15

**Authors:** Réiltín Werner, Amy Connolly, Michael Bennett, Collette K Hand, Louise Burke

**Affiliations:** 1 Pathology Department, Cork University Hospital, Cork, Ireland; 2 Department of Pathology, School of Medicine, University College Cork, Cork, Ireland

**Keywords:** Biomarkers, Tumor, QUALITY CONTROL, DNA, DIAGNOSIS, AUTOMATION

## Abstract

**Aims:**

Next-generation sequencing (NGS) is integral to the delivery of personalised medicine for targeted cancer therapy. Average turnaround times (TAT) from reference laboratories with advanced expertise in sequencing are typically 2–3 weeks. Prolonged TAT for biomarker analysis can adversely affect patient outcomes. The project aim was to establish an accredited NGS service integrated within a routine clinical diagnostic laboratory, in a designated tertiary cancer centre with no previous experience in NGS or bioinformatics.

**Methods:**

Platform selected was the novel Ion Torrent Genexus Sequencer with automated onboard library preparation, templating, sequencing and data analysis, with subsequent reporting using Oncomine Reporter software.

Entire workflow validation was performed with a targeted panel, the Oncomine Precision Assay, on formalin-fixed paraffin embedded clinical tumour samples. Oncomine Reporter software was used to report on variants including mutations, copy number variations and fusions across 50 key genes.

Samples included surgical resections, biopsies, cytology and commercial reference material. Assessment of criteria included analytical sensitivity, specificity, limit of detection, accuracy, repeatability and reproducibility, with the establishment of performance metrics and quality parameters.

**Results:**

High sensitivity, specificity and reproducibility were achieved. DNA/RNA input requirements optimised to >10 ng, and sequencing performance established with a limit of detection of 5% when depth of coverage of 2500X was reached. This NGS service attained ISO15189 accreditation with no non-conformances and >56% reduction in TAT.

**Conclusion:**

Successful implementation, clinical validation and accreditation of a novel NGS technology was achieved in this institution, with a significantly improved TAT of results to oncologists

**WHAT IS ALREADY KNOWN ON THIS TOPIC**

- This is a new concept, the implementation of an ISO15189 accredited next-generation sequencing (NGS) service in a clinical diagnostic pathology laboratory without any prior experience or specialised expertise in sequencing is not commonplace. Recent significant developments in NGS technologies, platforms and automated workflows have enabled this NGS naïve laboratory to establish an accredited, fully automated sample to report solution in-house.

**WHAT THIS STUDY ADDS**

- This study provides an NGS implementation roadmap for clinical diagnostic pathology departments that are facing challenges such as increased demands for advanced diagnostics via NGS, optimal turnaround times and accreditation requirements.

**HOW THIS STUDY MIGHT AFFECT RESEARCH, PRACTICE OR POLICY**

- Multigene molecular testing is now a fundamental part of cancer diagnosis. Its incorporation into the clinical diagnostic workflow allows for enhanced diagnostics, improvements in targeted treatments and cancer trials for patients, ensuring appropriate use of healthcare resources which will ultimately lead to improved outcomes for patients with cancer.

## Introduction

Oncological practises have undergone transformational changes over the past decade, having moved from a ‘one-size-fits-all’ approach, to now focusing on a more targeted therapeutic approach based on identified genomic variants.^1 2^ Molecular pathology techniques, and more specifically next-generation sequencing (NGS), are integral to the delivery of this personalised medicine approach.^3–5^ The rate of development of treatments, in addition to the rapid increase in demand for emerging novel types of biomarkers, has led to the selection of NGS rather than single platform assays as the preferred methodology for targeted analysis of tumour samples. Cork University Hospital, as a designated tertiary National Cancer Control Programme cancer centre servicing a population of approximately 1.4 million, has experienced a fivefold increase in requests for variant analysis testing in the last 5 years. There is increasing clinical demand for laboratories across Ireland, the UK, and beyond to integrate NGS and diagnostic molecular pathology reports into patient management workstreams.^1 6 7^ This places significant demands on the reference laboratories with advanced expertise in sequencing. The target turnaround time (TAT) from these referral centres is typically more than 3 weeks according to a recent report from European Society for Medical Oncology.^8^ Prolonged TAT for biomarker analysis can adversely affect patient outcomes and early and consistent access to molecular testing is of critical importance.^9 10^ It is imperative that any integration of NGS services must achieve the best possible TAT, and meet best practice guidelines.

In line with requirements for compliance with national and international guidelines^11–14^ and to provide an optimal service for the patients of the region, a proposal to establish an ISO15189 accredited NGS service integrated into this clinical diagnostic pathology laboratory was implemented.

The absence of onsite experience in NGS poses challenges in implementation with some of the most common barriers being^15^:

- Complexity; user expertise in sequencing including bioinformatics.
- Tissue requirements; limited availability of sample volume.
- Cost per assay; penalty for small sample numbers.

However, many of these challenges have been decreased by the rapid and significant developments now available in NGS technologies and platforms, which have enabled laboratories such as this one to implement the technology with improved throughputs and increased cost-effectiveness.^16^ In this study, we set out to introduce a novel, fully automated NGS workflow, with a sequencing TAT (24 hours) currently not attainable in this country. The Ion Torrent Genexus is recognised as the first ‘turnkey’ NGS solution that streamlines the workflow and can deliver sequencing results in a single day,^17^ it was selected as the solution for this laboratory as it addresses the barriers listed above with an automated specimen to report workflow. The Genexus does not require a high level of user expertise in bioinformatics and reporting; it facilitates a minimal nucleic acid input volume of approximately 10 ng, it has a multiplexing capability of up to 32 library preparations in a single run while also allowing run sizes of 2–4 samples using a cost per assay model instead of cost per run.

In a diagnostic setting, optimal clinical validation is paramount^18^ and was a principal component of this NGS project. Ensuring adherence to Irish National Accreditation Board standards (INAB ISO15189:2012) and available best practice guidelines from the Association for Molecular Pathology/College of American Pathologists (AMP/CAP)^19^ was essential.

To define and understand required assay parameters and validation protocols in advance of implementation, a comprehensive literature review was conducted to examine and evaluate the different methods of analytical validation for NGS in use across molecular laboratories nationally and internationally. This resulted in the identification of relevant research articles, detailing the utilisation of formalin-fixed paraffin wax embedded (FFPE) tumour tissue samples subjected to orthogonal testing in combination with commercial control material.^16 20–26^ This approach is in line with guidelines from the AMP/CAP,^19^ and therefore, provided the basis for the comprehensive NGS validation strategy employed. The project plan was defined using industry-standard Lean Six Sigma tools^27^ such as process maps, project impact statement, SWOT analysis and Gantt charts.^28^

In this report, we describe how the first clinical diagnostic pathology laboratory across the UK and Ireland implemented a fully automated accredited NGS service without any prior experience in NGS technologies.

### Objectives

The following key objectives were identified:

1. Optimisation and verification of methodology and platforms for entire sequencing workflow, including sample preparation, and sequencing using a targeted oncology panel; the Oncomine Precision Assay (OPA), with subsequent bioinformatics analysis on the Ion Torrent Genexus sequencer, and Oncomine Reporter software enabled reporting.
2. Attain full accreditation status (INAB ISO15189:2012) for NGS service.
3. Reduce TAT for NGS results.

## Materials and methods

The protocol assessed the performance of a targeted oncology panel (OPA) for hotspot genes, copy number variations (CNV) and gene fusions for clinical application in solid tumour testing in a controlled and phased manner.

### Sample selection

**Figure 1:**
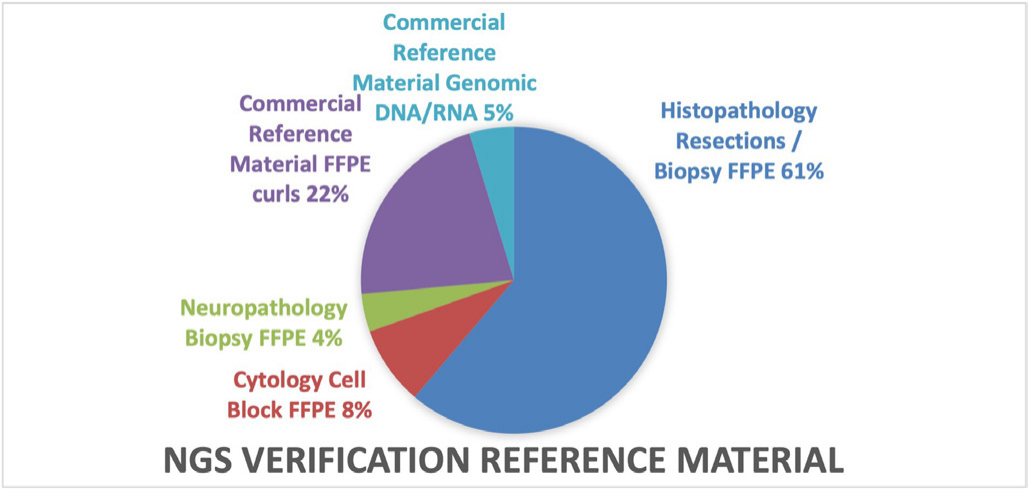
Range of NGS verification reference material spanning multiple specimen types. NGS, next-generation sequencing.

NGS validation was performed across multiple tests (n=276) on the Genexus. This validation was performed on clinical tumour samples previously characterised via an orthogonal NGS assay externally or accredited PCR methods onsite. Anonymised real-world samples (n=203) were used (figure 1) comprising NSCLC, colorectal cancer, melanoma, sarcoma, breast, brain, liver, urothelial and cervical cancers processed from a range of specimen types including surgical resections and biopsies (n=169), cytology cell blocks (n=23) and neuropathology biopsies (n=11).

Preanalytical evaluation of sample suitability was carried out prior to sample inclusion in the validation. For each paraffin block, 3 µm sections were cut using a semiautomated Leica Rotary Microtome (RM2255) onto a glass slide and stained using routine H&E staining. The slides were anonymised and assessed for neoplastic cell content (NCC) by a pathologist.

NCC ranged from 10% to 90% with macrodissection used for enrichment of samples to above an assay-specific minimum threshold of 20% or optimally to 50% tumour cell content. In the samples with suboptimal NCC, areas of interest (AOI) with a concentration of tumour cells were marked on the H&E-stained slide. The AOI was matched to unstained slides and scraped into sterile micro-centrifuge tubes for sample preparation, thereby reducing any sample dilution from normal and inflammatory cells.^29^

### Reference materials and commercial controls

Assay validation also included the utilisation of reference cell lines, genomic DNA/RNA and reference tissue material for evaluation of assay performance. These reference materials are controls that are homogenous and well established for the calibration/validation of diagnostic instruments.^30^

Commercial control material was procured from External Quality Assessment organisations, such as GENQA, EMQN, QUIP and commercial suppliers (table 1).

**Table 1:** Commercial controls included in study tested with OPA over 20 runs

Manufacturer	Reference name	Product code
HORIZON	ALK /ROS/RET	HD784
HORIZON	EGFR	HD300
HORIZON	KRAS	HD301
HORIZON	MULTIPLEX	HD789
HORIZON	ONCOSPAN	HD832
ACROMETRIX	HOTSPOT ONC	969 056
SERASEQ	RNA FUSION	0710–0496
SERASEQ	NTRK RNA	0710–1031

OPA, Oncomine Precision Assay.

### Sample preparation

Samples were deparaffinised in xylene (5 min), washed in 100% ethanol (2×5 min) and air-dried for 15 min. Tissue lysis was performed as per manufacturer instructions (Ion Torrent Genexus FFPE Combo kit) with Proteinase K in heat-blocks at 55ºC and 90ºC for 1 hour each prior to nucleic acid extraction. This off instrument pre-processing of samples took approximately 3 hours.

Following routine FFPE lysis protocol to obtain appropriate starting material, two methods of extraction were verified. The first was a semiautomated extraction with the KingFisher DuoPrime instrument with MagMAX FFPE DNA/RNA Ultra kit (Thermo Fisher, Scientific); the DNA/RNA concentration (n=181) was determined manually by fluorometric quantitation using the Qubit 2.0 Fluorimeter with Qubit DNA dsDNA BR Assay and RNA BR Assay kits (Reagecon).

The second extraction method streamlined the purification workflow with full automation using the Genexus Purification Instrument (GPI) on DNA and RNA over four runs (n=95). The hands-on time (10 min) was minimal, and the GPI extracted both DNA and RNA sequentially (Ion Torrent Genexus FFPE DNA/RNA Purification Combo Kit). The GPI has a built in Qubit for a fully automated workflow.

Quantified nucleic acids, with a minimum of 10 ng nucleic acid input, were prepared in an OPA output plate and manually transferred directly onto the Genexus Integrated sequencer.

### Library preparation, sequencing and data analysis

OPA is a targeted pan cancer panel encompassing 78 variants, across 50 key genes, including mutations (45), CNVs (14), fusion variants (19) and hotspot mutations (substitutions, insertions and deletions) (table 2).

**Table 2:** Oncomine Precsion Assay content

DNA hotspots			CNV	Fusions	
AKT1	ESR1	MAP2K2	ALK	ALK	NTRK2
AKT2	FGFR1	MET	AR	BRAF	NTRK3
AKT3	FGFR2	MTOR	CD274	ESR1	NUTM1
ALK	FGFR3	NRAS	CDKN2A	FGFR1	RET
AR	FGFR4	NTRK1	EGFR	FGFR2	ROS1
ARAF	FLT3	NTRK2	ERBB2	FGFR3	RSPO2
BRAF	GNA11	NTRK3	ERBB3	MET	RSPO3
CDK4	GNAQ	PDGFRA	FGFR1	NRG1	AR
CDKN2A	GNAS	PIK3CA	FGFR2	NTRK1	EGFR
CHEK2	HRAS	PTEN	FGFR3		
CTNNB1	IDH1	RAF1	KRAS		
EGFR	IDH2	RET	MET		
ERBB2	KIT	ROS1	PIK3CA		
ERBB3	KRAS	SMO	PTEN		
ERBB4	MAP2K1	TP53			

All samples DNA and RNA (n=276) were processed on the Genexus with automated library preparation, sequencing and bioinformatic analysis (Genexus software V.6.3 Thermo Fisher Scientific).

### Reporting

Following sequencing by the Genexus, data were uploaded to an Oncomine Reporter (OR) software (V.5.5 Thermo Fisher, Scientific). This is a genomic analysis tool developed specifically for downstream analysis of NGS data and the generation of complete reports, including the stratification of variants, and recommendations for therapy and clinical trials. The database is monitored, with software updated monthly following review of labels guidelines (NCCN, EMA, Food and Drug Administration)^14 31 32^ and changes to available clinical trials. VCF (variant call files) from the Genexus were uploaded to the OR software, and reports generated for authorisation by the consultant pathologist.

### TAT measurements

TAT was established as, request date to complete molecular report date, defined as a consultant pathologist authorised, integrated report of NGS results, with corresponding Immunohistochemistry results, visible in the electronic medical record to the treating clinician. TAT was calculated in business days.

### Accreditation to ISO15189

An application was made to the INAB to add this NGS service for FFPE samples to the annual accreditation assessment schedule. It was audited to ISO15189 standards by the external body following validation.

### Analytical validation

To address compliance and audit against standards, laboratory-specific performance metrics were established for the DNA/RNA targeted assays including, limits of detection (LOD), measurement uncertainty, minimal depth of coverage, minimum read counts, mapped reads, uniformity and variant allelic frequency.

The OPA panel was assessed to determine positive percentage agreement (PPA) and positive predictive value (PPV) for each variant type. Assessment parameters included determining analytical sensitivity, specificity, LOD, accuracy, reproducibility, interlot, interoperator and inter-run variability.

### Establishment of quality performance metric

The number of assays (n=276) enabled the accurate establishment of test performance characteristics. Like previous studies^18^ the quality and depth of coverage metrics were measured across all clinical validation specimen data sets to establish acceptable run-level quality control parameters. These performance metrics included the percentage of reads mapped to the reference sequence for the DNA and RNA libraries. A minimum threshold of each performance metric was established for ongoing quality control.

## Results

**Figure 2:**
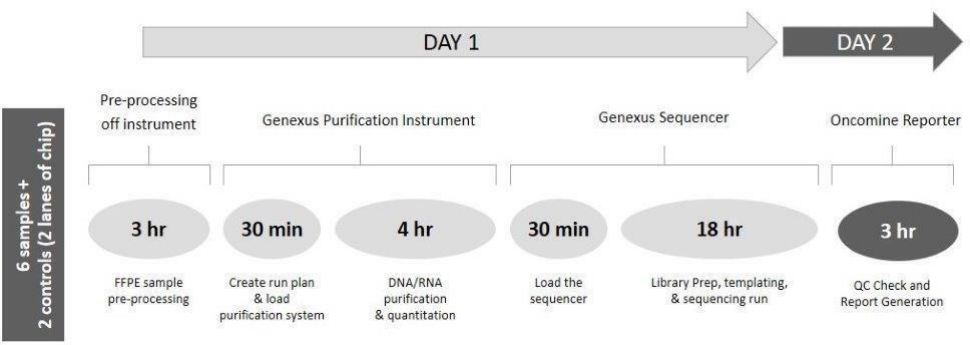
NGS processing workflow established in-house with 2- day TAT and minimal hands-on-time. NGS, next-generation sequencing; TAT, turnaround time.

This is a multifaceted project carried out over 12 months from initial concept to ISO15189 accreditation. During the overall verification period, 276 samples underwent NGS testing as per the routine sample processing to report workflow (figure 2).

### Nucleic acid extraction and quantification

**Figure 3:**
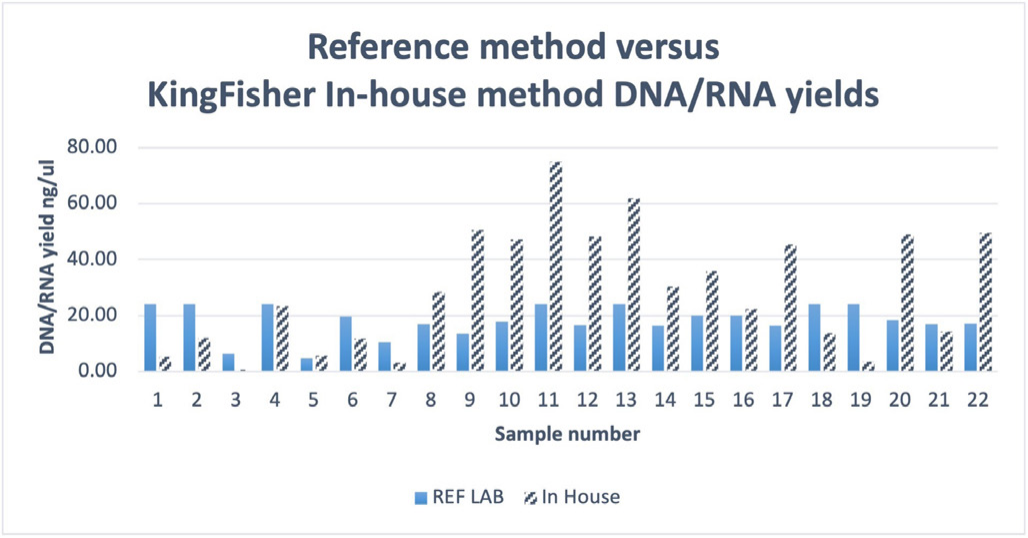
Sample DNA: RNA yield comparison of Reference method versus kingfisher.

Twenty-two samples previously characterised by an accredited reference method were re-extracted with the MagMAX kit on the KingFisher DuoPrime. Minimum input requirements of 10 ng of DNA/RNA were obtained for running on the Genexus sequencer, 100% of samples included were above this threshold and concordant with orthogonal testing when sequenced onsite. The average nucleic acid yield obtained increased from 18.13 ng/μL to 28.95 ng/μL when performed in-house (figure 3). A total of 181 extractions were performed with this method and tested with the OPA on the Genexus sequencer.

**Figure 4:**
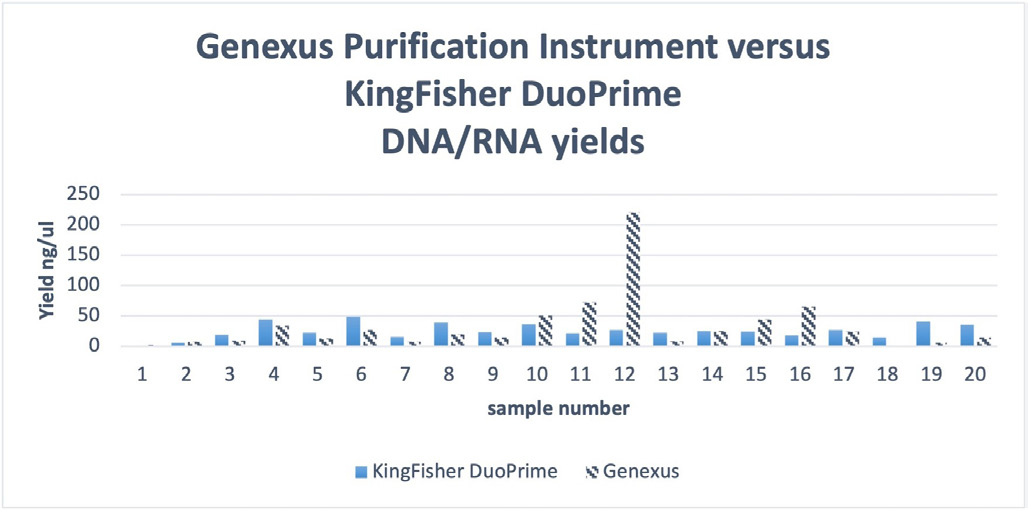
Sample extraction and purification DNA:RNA yield comparison of Genexus and KingFisher DuoPrim.

The validation of extractions of DNA/RNA on the GPI also gave comparable results. Extraction and purification of samples (n=96) run on the GPI were sequenced on the Genexus system. Where data on previous extraction yields were available (n=20) a comparison was made, the average yield increased from 25.4 ng/μL to 33.1 ng/μL (figure 4).

Minimum input requirements for OPA NGS were assessed with an input from the purification instrument as low as 10 ng DNA/RNA yield resulting in a profile consistent with orthogonal methods. However, when input was increased to >20 ng the overall metrics of the run improved with higher reads per lane.

### Sequencing performance

Run success was based on passing performance metrics established over the course of the optimisation. In total, data on over 250 assays (FFPE tissue and controls) were collated from the Genexus software, enabling the generation of performance metrics specific to this centre. These metrics facilitate the determination of which findings are released when curating reports (table 3).

**Table 3:** Performance metrics established at optimisation

FFPE OPA performance metrics	
Metric	Target
Final reads	10–12M
Raw read accuracy	98–99%
% Loading	87%–92%
Enrichment	99.90%
Library	99.90%
Mapped reads/DNA library	> 500K (>800K for 5% LOD)
Mapped reads/RNA library	> 100K
% Reads on target	>90%
Base coverage depth	>1000 (>2500 for 5% LOD)
Uniformity	97%–99% (>90% for 90%)
End-to-end reads	>90%
Reads/amplicon	>500
AF	> 5%/0.05
MAPD	>0.5 (0.18–0.24)
RNA detection	>5/7
Mean AQ20 read length	85–95
Mean read length DNA	85–100
Mean read length RNA	70–100
Base call accuracy	97%–99%

AF, allelic fraction; LOD, limits of detection; MAPD, median absolute pairwise difference.

One of these metrics is variant allelic fraction (VAF), which corresponds to the fraction of sequencing reads harbouring the mutation. AF is influenced by the proportion of tumour cells in the sample, the presence of copy number alterations but also, most importantly, by the proportion of cells within the tumour that carry the mutation.^33^

### OPA performance: variant detection accuracy

The assay verification material included 203 real-world samples supplemented by controls which spanned 189 variants detected across 45 key genes in the OPA panel. There was no material available to detect *AKT2/3, ARAF, CDK4* and *CHEK2*. The 11 currently reportable or actionable variants for non-small cell lung cancer (NSCLC) were extensively verified (n=126) (*ALK, EGFR, BRAF, KRAS, NTRK 1/2/3, ROS, RET, MET, ERBB2*).

The real-world samples included two samples which failed the minimum DNA/RNA input requirements (<0.07 ng/μL and 1.24 ng/μL); these were flagged as fails by the software, but they were concordant with orthogonal results. Four colorectal samples failed performance metrics due to prolonged fixation, inappropriate storage and degradation, and were removed from the comparative analysis. True positive, false positives and false negatives were determined for each sample for the targeted regions meeting the minimum quality requirements. The overall concordance to orthogonal methods with PPA and PPV in this validation is >99%.

### OPA analytical reproducibility, sensitivity and specificity

**Figure 5:**
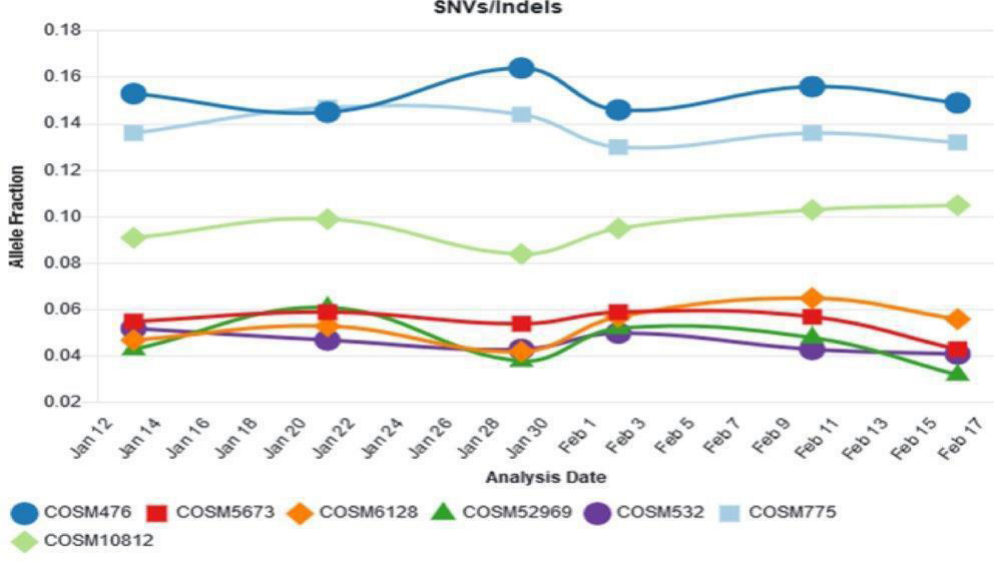
HD789 control reproducibility assays across seven variants indicating allele fraction within specifications.

The small DNA variant analytical specificity was assessed by correctly identifying samples that do not harbour any of the variants being profiled. A range of samples with confirmed non-detected results (n=42) were concordant with orthogonal methods and therefore 100% specific. Good reproducibility was demonstrated by performing sequencing on control material across six runs with different operators, reagents and chips on different days with all results consistent and within range (figure 5).

Twenty samples previously validated by an orthogonal method were tested for RNA fusions. All cases were correctly identified conferring an assay sensitivity of 100%. In line with expected results, 47 fusions across all samples were detected conferring 100% specificity. Intrarun and inter-run reproducibility was assessed with 3 replicates of an SLC34A2(4)-ROS1(34) positive NSCLC sample and demonstrated 100% concordance between and within runs.

In addition to the real-world samples with a sensitivity of >99%, commercial reference samples (n=56) were used over 20 runs at varying dilutions, which allowed the determination of LOD across multiple variants. While VAFs down to 0.01 were detected, a performance metric cut-off of 5% or 0.05 LOD was established when a depth of coverage of 2500X was reached.

**Figure 6:**
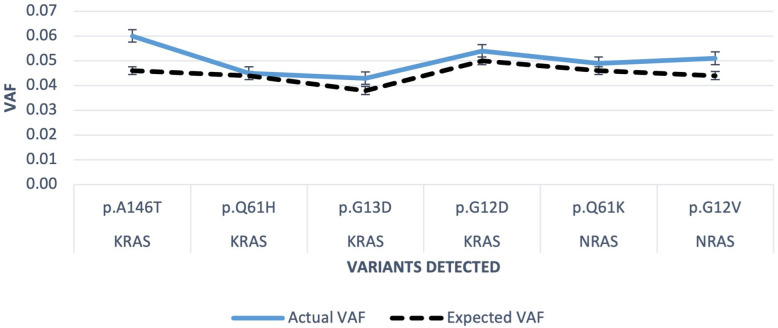
Run 15 HD301 KRAS multiplex FFPE DNA reference standard. FFPE, formalin-fixed paraffin wax embedded; VAF, variant allelic fraction.

Figure 6 demonstrates *KRAS* Quantitative Multiplex FFPE Standard HD301 AF for each variant detected versus expected VAF.

**Figure 7:**
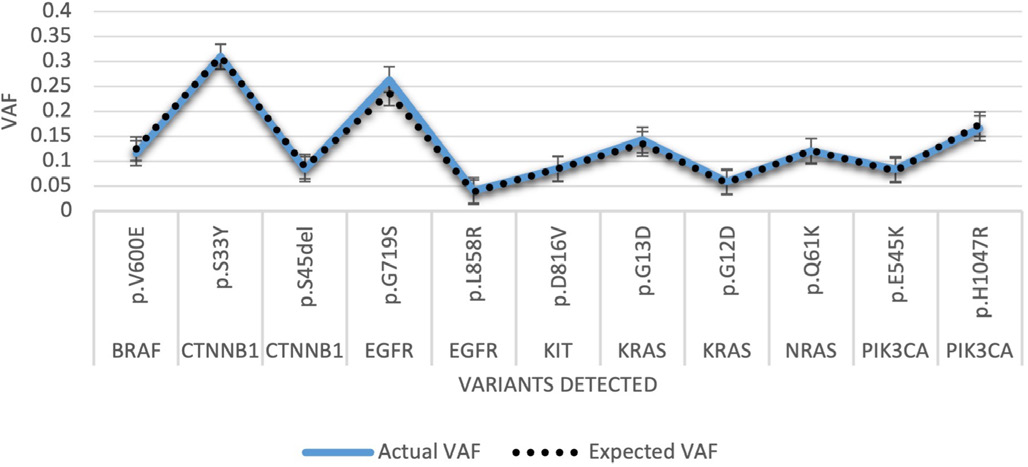
Run 18 HD832 Oncospan reference standard. VAF, variant allelic fraction.

The Oncospan DNA Reference Standard HD832 in figure 7. depicts further concordance, the AF for each variant detected versus expected VAF.

**Figure 8:**
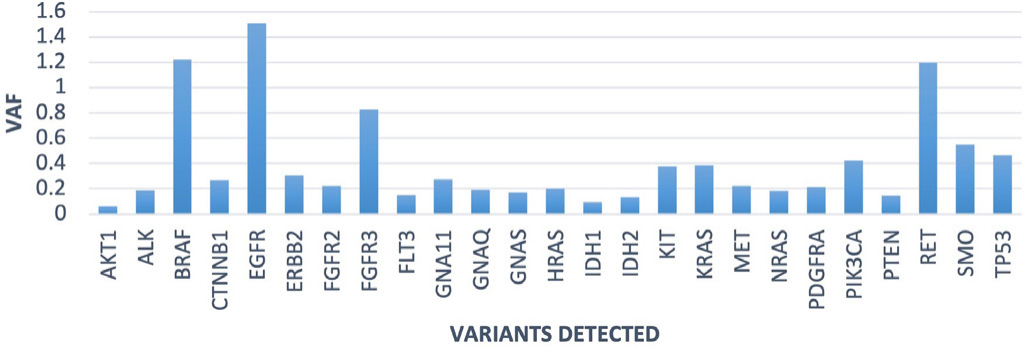
Run 10 AcroMetrix reference standard, variants detected and allelic fraction.

The AcroMetrix Oncology Hotspot control also gave 100% specificity and 100% sensitivity across genes detected in the OPA (figure 8).

### NGS results TAT

Six months postimplementation an audit of NGS reports in-house gave a 7-day average TAT, compared with the target TAT of the outsourced reference laboratory of 15 working days, with an additional day for authorisation by the consultant pathologist. Implementation of this system resulted in a 56% reduction in TAT of NGS results.

### Accreditation

The INAB audit comprised a thorough examination of the NGS service considering each of the (ISO15189) standards. The INAB assessment was successful with no non-conformances.

## Discussion

Technological evaluations resulted in the selection of the novel Ion Torrent Genexus sequencer and OPA as the appropriate NGS solution for this hospital. The Genexus system was chosen as it did not require user expertise in bioinformatics and reporting; it facilitated a minimal sample input volume and flexible run sizes with a cost per assay model instead of cost per run. The Genexus streamlines the workflow and can deliver sequencing results in a single day, while meeting national and international guidelines and standards.^17^

The OR software was successfully integrated allowing NGS reporting without the need for bioinformatics expertise. Bespoke report templates were curated and aligned to local requirements, thus facilitating the linking of variants to labels guidelines and clinical trials.

The service also successfully attained ISO15189 accreditation and significantly improved TAT for cancer patients in this region.

## Conclusions

A collaborative approach was adopted for this project, with continuous dialogue between consultant pathologists, oncologists, scientists and administrative staff to enable a seamless transition to the new workflow. Implementing this NGS technology has enabled accurate on-site testing of multiple cancer genes in a single specimen, ensuring an optimal service for cancer patients. As a designated tertiary cancer centre this project facilitated the development of a service that can maintain pace with the expansion of identified actionable genetic mutations as defined by international guidelines.^14^

Molecular testing is a fundamental part of cancer diagnosis. Its incorporation into our clinical diagnostic workflow allows for enhanced diagnostics, improvements in oncological possibilities and cancer trials for patients, ensuring appropriate use of healthcare resources which will lead to improved outcomes for cancer patients. The Ion Torrent Genexus system allows a wider implementation of NGS for molecular profiling, bringing precision medicine mainstream.

## Data Availability

All data relevant to the study are included in the article.
